# Innovative finding of 266-nm laser regulating CD90 levels in SDSCs

**DOI:** 10.1038/s41598-021-93508-x

**Published:** 2021-07-06

**Authors:** Ray-Ling Hsiao, Yen-Chung Chen, Mei-Yue Huang, Chiang-Yun Chen, Yu-Wei Lin, Chung-Yi Wu

**Affiliations:** 1 Ph.D. Program for Cancer Biology and Drug Discovery, China Medical University and Academia Sinica, Taichung, Taiwan, ROC; 2 Academia Sinica, Taipei, Taiwan, ROC; 3 Maria Von Med-Biotechnology Co., Ltd, Taipei, Taiwan, ROC; 4 Mainz Dermatologic Clinic, Taipei, Taiwan, ROC; 5 Genomics Research Center, Academia Sinica, Taipei, Taiwan, ROC

**Keywords:** Cell biology, Molecular biology

## Abstract

We used light to irradiate skin-derived stem cells and tried to find any cellular protein alterations 24 h after illumination. A 266-nm laser with four intensities was used, and of the nine cell markers that were surveyed in our trials, only CD90 was downregulated at an intensity of 20 μJ for 10 s. Repeated illuminations from the 266-nm laser at seven intensities revealed that CD90 expression was downregulated 14.6–28.8%, depending on light intensity. The maximal effect was noted at an intensity of 30 μJ for 2 s. This innovative finding reveals that a 266-nm laser can regulate protein expression in skin-derivative stem cells.

The fact that nucleotides can absorb light energy was reported in 1963. Two ranges of wavelengths were studied, one at 180–240 nm and the other at 240–300 nm^1^. A report that cellular DNA is damaged by 193-nm light persuaded us to avoid the first range^2^. Additionally, the absorption peak for double and single strand DNA is around 260 nm^3^. We surveyed the absorption spectrum of each nucleotide over the range of 240–290 nm (Fig. 1). This inspired us to use a 266-nm laser to irradiate skin-derived stem cells (SDSCs) and survey their responses.

**Figure 1:**
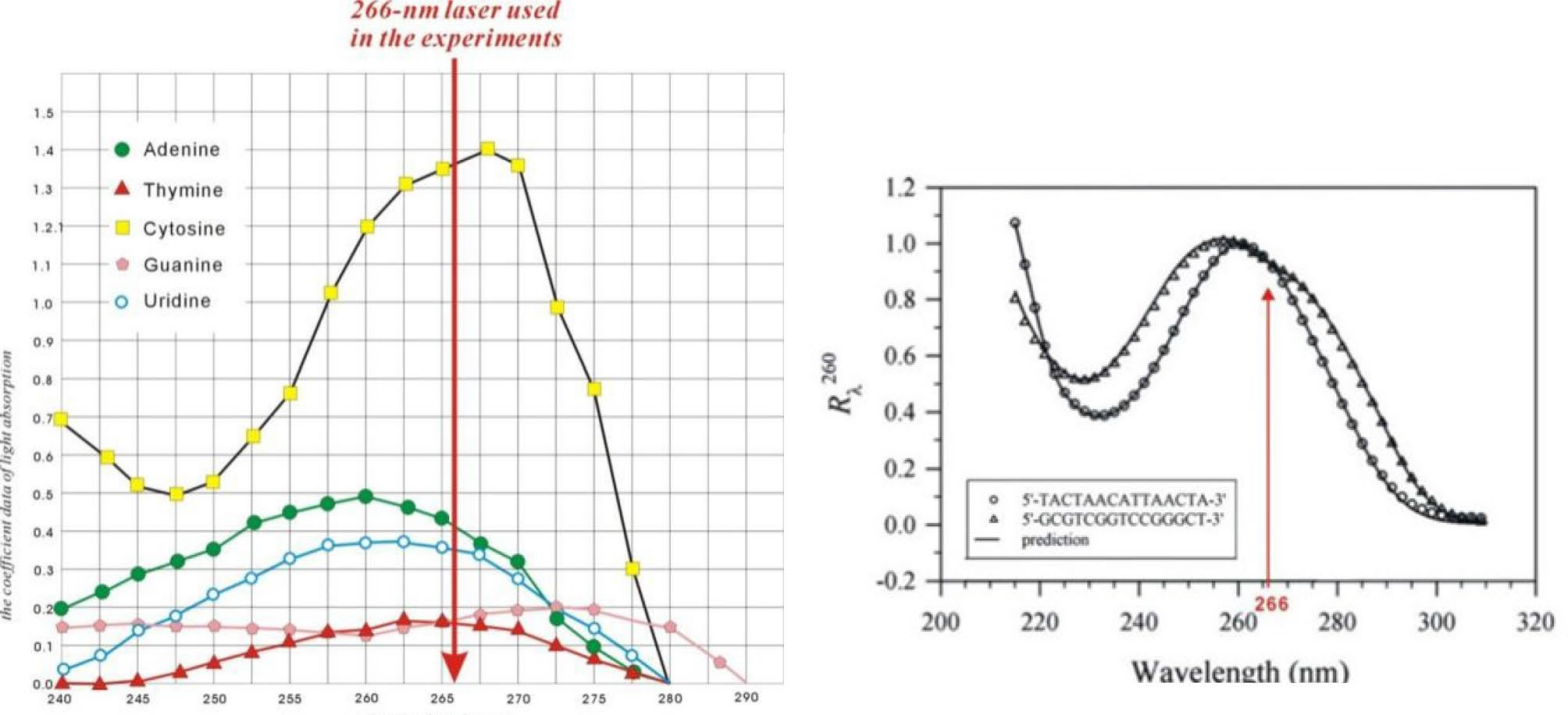
Absorption spectra of nucleotides and comparison of predicted (solid lines) and experimentally measured (symbols) spectra for double and single-stranded DNA oligomers. The left part of the figure, the x-axis shows the wavelength in nm and the y-axis shows the coefficient data of light absorption. The light from the 266-nm laser is indicated with a red arrow and presented in the nucleotide absorption spectrum. The image is from the assay of the absorption spectra of 50 μM/L of each nucleotide. The right part of the figure, the GC content in the sequences is 20% (open circle) and 80% (open triangle). The image was modified from Tataurov et al.^3^.

That light can alter proteins has been known; however, those studies focused on wavelengths from 312–750 nm, as described in a review article by Arnaud Gautier in 2014^4^, but there have been no studies regarding the effect of 266-nm light. Here, we report an innovative finding that 266-nm light could regulate the expression of CD90 in SDSCs. The interesting difference between light at 266 nm versus 312–750 nm is that 266 nm is located in the absorbance range of nucleotides, and double or single stranded DNA, 266 nm is not in the range of protein absorption^5^.

Lasers with different wavelengths have potential use in cancer therapy. Laser illumination by light in the 630–762-nm range could kill cancer cells^6^. Based on previous studies described by Gautier in 2014^4^, Patrizia Agostinis in 2011^6^, and Tiina Karu in 1993^7^, we believe that the wavelength is a major property for light to have a specific effect.

A series of studies of 266-nm light has been reported. In 1993, Tiina Karu described the high-repetition-rate pulse of 271-nm light (HRRP-271), the continuous wave of 270-nm light (CW-270) from a copper laser, and powerful picoseconds pulse 266-nm (PPP-266) light from an Nd-YAG laser. Karu irradiated HeLa cells for 3 h and noted that the cellular population increased in the HRRP-271 and PPP-266 groups. An increased DNA replication rate was also noted in PPP-266 and HRRP-271^7^. The goal of those studies was to find a way to kill the cancer cells (HeLa cells), but the results disappointedly indicated that some cancer cells grew better than control, and some cancer cells showed increased DNA replication^7^. Those studies were not followed up on. Our group has tried to continue studying the 266-nm laser effect on SDSCs. The light used in our experiment was also generated from an Nd-YAG laser, but intensities were quite different from Karu’s. The laser was set at a rate of 10 pulses per second, the power strength from 5 to 40 μJ, and the exposure time from 2–10 s. Karu’s experiments set the pulse rate in thousands per second, power strength in mJ, and the exposure time in hours. Karu’s purpose was to kill the cancer cells, but ours was to investigate how the 266-nm laser acted on protein expression of stem cells.

This is the first time a 266-nm laser has been used to investigate regulation of protein expression in SDSCs. Initially, we chose nine cell markers to track after irradiating SDSCs for 2 to 10 s, followed by culturing for 24 h. CD90 was the only marker found to be obviously downregulated under these conditions, and the other eight markers had no remarkable change. In this experiment, we set four intensities of the laser: 5 μJ for 2 s, 5 μJ for 10 s, 20 μJ for 2 s, and 20 μJ for 10 s. Only 20 μJ for 10 s generated an obvious response (Fig. 2). The laser power (P) and exposure time (T) would be two major factors in the energy (E) the cells were exposed to, and too low or too high E would not produce measurable responses.

**Figure 2:**
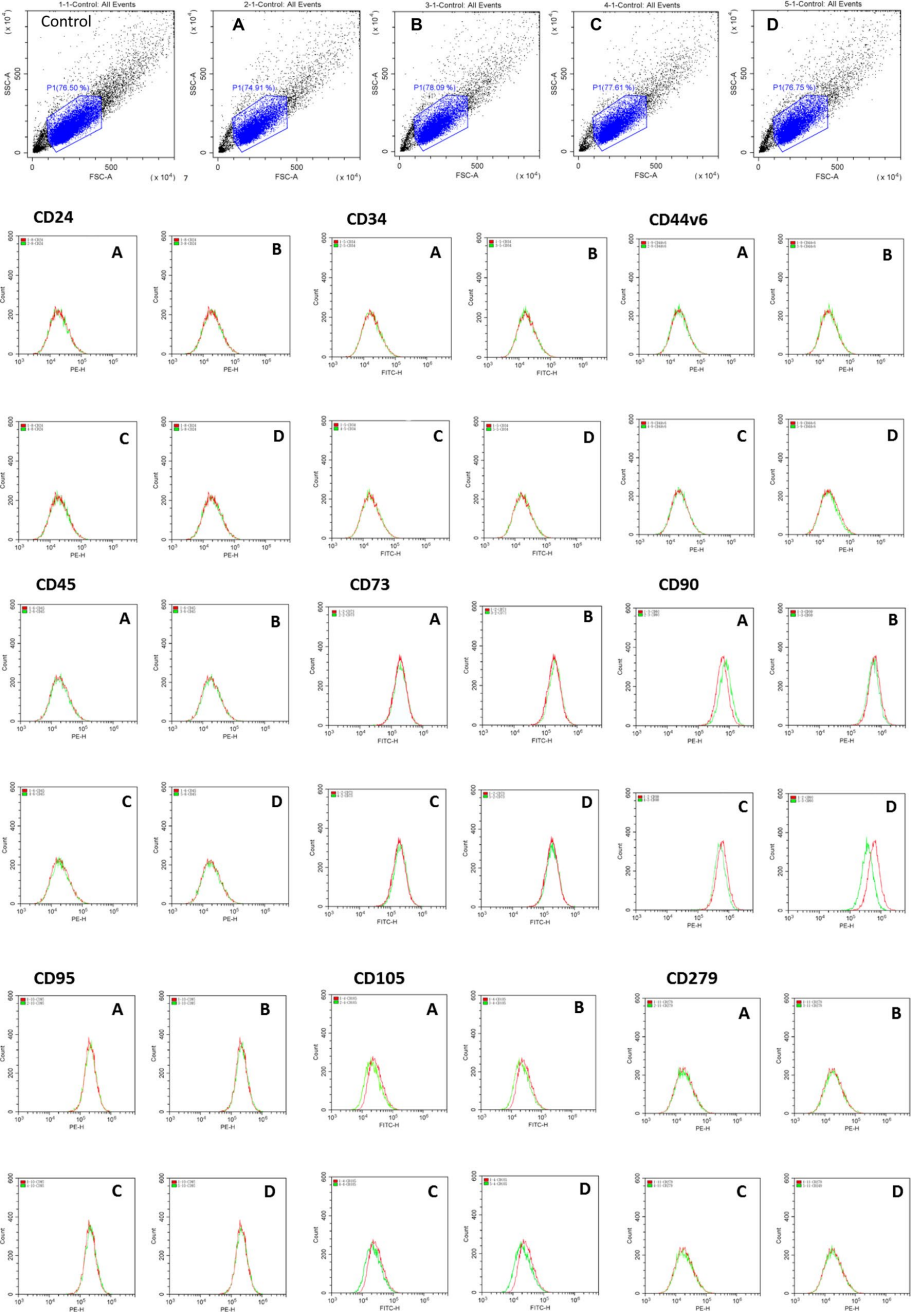
Fluorescence intensity curves of nine cellular markers in skin-derived stem cells (SDSCs). Nine cellular markers (CD24, CD34, CD44v6, CD45, CD73, CD90, CD95, CD105, and CD279) were screened to detect alterations in SDSCs after laser irradiation at four different intensities: (**a**) 5 μJ for 2 s; (**b**) 5 μJ for 10 s; (**c**) 20 μJ for 2 s; and (**d**) 20 μJ for 10 s. The upper portion of the figure indicates the positive cells in the indicated region. In the lower portion of the figure, the red line represents the control cells and green line corresponds to laser-irradiated cells. The x-axis indicates the intensity of fluorescence and the y-axis denotes the cell count. Treatment D considerably downregulated CD90 levels.

When we repeated these experiments with the 266-nm laser intensities as 5 μJ for 2 s, 20 μJ for 2 s, 20 μJ for 10 s, 30 μJ for 2 s, 30 μJ for 10 s, 40 μJ for 2 s, and 40 μJ for 10 s, the downregulation of CD90 varied from 14.6–28.8% according to the intensity (Fig. 3, Supplementary Table 2). The factors P and T appeared to be related to protein expression alteration, and we have tried to find a formula describing these data. Although the protein expression was altered, the cellular morphology and number of SDSCs were not appreciably changed 24 h after illumination (Supplementary Fig. 1, Supplementary Table 3).

**Figure 3:**
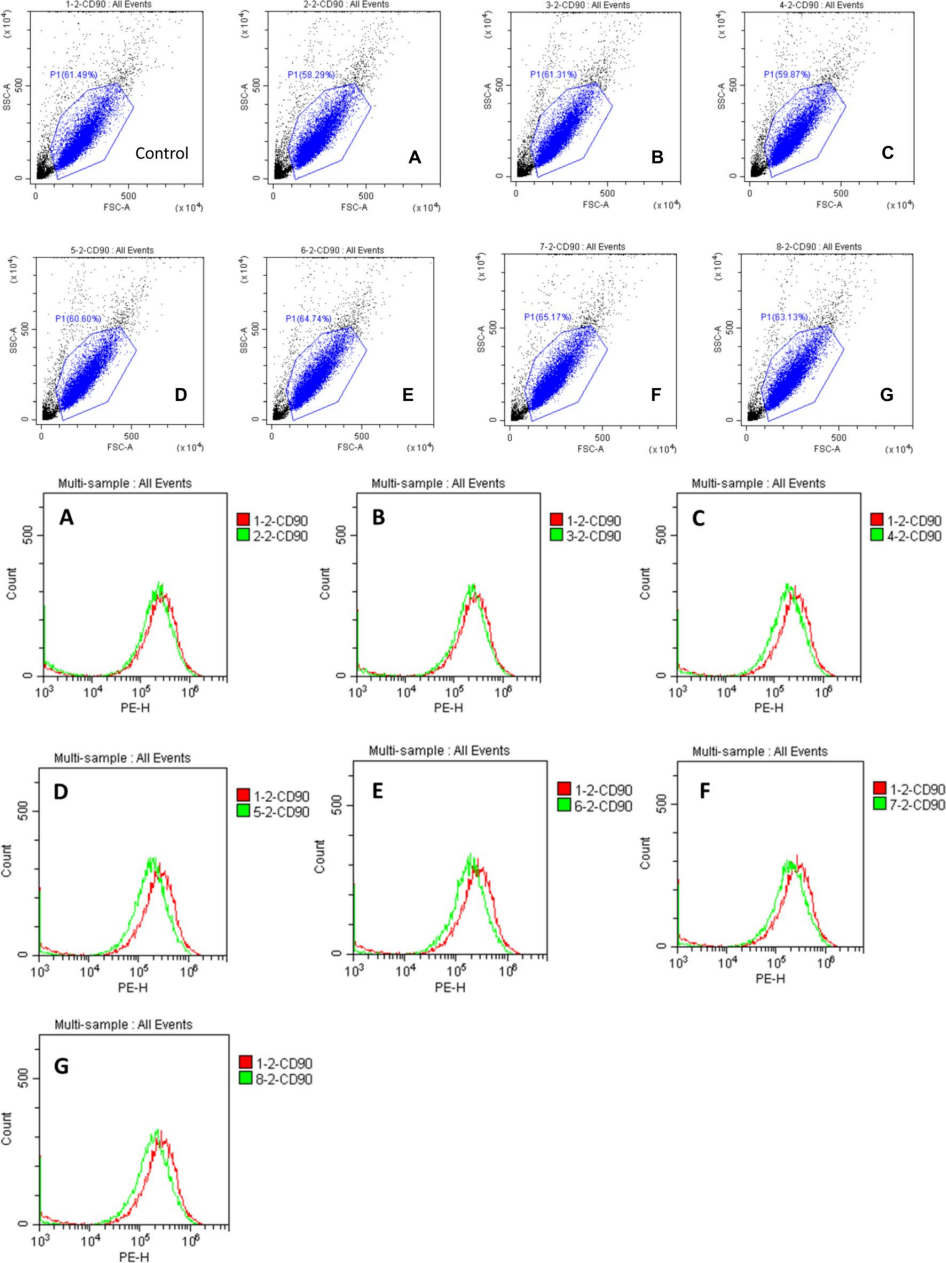
Fluorescence curves of CD90 in skin-derived stem cells (SDSCs) subjected to treatment with seven laser intensities. (**a**) 5 μJ for 2 s; (**b**) 20 μJ for 2 s; (**c**) 20 μJ for 10 s; (**d**) 30 μJ for 2 s; (**e**) 30 μJ for 10 s; (**f**) 40 μJ for 2 s; and (**g**) 40 μJ for 10 s The upper portion of the figure indicates the positive cells in the indicated region. In the lower portion, the red line represents the control group and green line represents the experimental group. The x-axis indicates the fluorescence intensity and the y-axis denotes the cell count. The data are shown in Supplementary Table 2.

We next tried to irradiate A549 lung cancer cells, which express CD90, to see whether a similar effect could be observed. No downregulation could be observed after the same experimental procedures (Supplementary Fig. 2), which might mean the effect on CD90 was cell specific and not protein specific.

The specific findings from our study were that CD90 expression was downregulated upon exposure to 266-nm light but not the other markers we investigated. This effect was consistently repeatable, meaning the effect of the light irradiation in the cells was specific and causal. Such characteristics indicate the potential to use a laser to regulate protein expression and even cellular function in the future. We reported laser power strength, pulse width, and exposure time, which are far different from Karu’s reports^7^. Although this study demonstrated that light could regulate protein expression in SDSCs, further investigation is needed to determine a potential practical use.

## Results

### Absorption spectra of nucleotides

The absorption spectra of nucleotides were calculated based on the Beer–Lambert Law^8^:

$$ Absorption{\text{ }} = {\text{ }} - {\text{ }}log10(transmittance/100). $$

A wavelength range of 240–290 nm was used to detect the absorption of nucleotides; the results are shown in Fig. 1.

### Effect of 266-nm laser on nine cell markers on SDSCs

To detect changes in SDSC protein expression after irradiation, nine cellular markers (CD24, CD34, CD44v6, CD45, CD73, CD90, CD95, CD105, and CD279) were evaluated (Supplementary Table 1). The cells were illuminated with a 266-nm laser at four different intensities (5 μJ for 2 s, 5 μJ for 10 s, 20 μJ for 2 s, and 20 μJ for 10 s). SDSCs were cultured for 24 h, and flow cytometry was performed to quantify protein expression changes. The only clear alteration was in CD90 expression, which was downregulated at 20 μJ for 10 s (Fig. 2).

### Repeated determination of CD90 expression in SDSCs exposed to different intensities of light from a 266-nm laser

To determine the cause of the changes in CD90 expression after laser irradiation, SDSCs were subjected to 266-nm laser irradiation at seven intensities (5 μJ for 2 s, 20 μJ for 2 s, 20 μJ for 10 s, 30 μJ for 2 s, 30 μJ for 10 s, 40 μJ for 2 s, and 40 μJ for 10 s). These results revealed that CD90 expression was downregulated (14.6–28.8%) after 24 h (Fig. 3, Supplementary Table 2). CD90 expression differed according to the intensity of light applied. The cell morphology and number were evaluated by optic microscopy, which revealed no obvious changes (Supplementary Fig. 1, Supplementary Table 3).

### A549 cells did not show significantly altered CD90 expression after 266-nm laser irradiation

A549 cells were exposed to a 266-nm laser in two experiments. Initially, the three different intensities used were 5 μJ for 2 s, 30 μJ for 2 s, and 70 μJ for 5 s. The second trial used 30 μJ for 3 s and 100 μJ for 10 s. There was no significant change in CD90 expression under these conditions (Supplementary Fig. 2).

## Discussion

Light in nature is important for various biological processes, such as photosynthesis of plants, blooming of flowers, ripening of fruits, phototropism of small insects, and optic vision in humans and animals. Light at different wavelengths would have different effects^4,6,7^. Light can enter cells and alter structure, charge, and functions of proteins; this can then influence other cellular functions^4^. Significant work has focused on wavelengths around 315–750 nm^4^, which is in the range of visible and near UV light. However, 266-nm light has rarely been studied, perhaps because this wavelength is not in the range absorbed by proteins^5^. Moreover, 266 nm is in the range of nucleotide and DNA absorption spectrum^1,2^ (Fig. 1).

Nucleotides absorb at 180–240 nm and 240–300 nm, as reported by Voet D. in 1963^1^. We selected a 266-nm laser because 193-nm light was reported to damage cells and DNA^2^. We used laser light because it is a mono wavelength light and can have its specific effect. The 266-nm laser was quadruple from 1064-nm of Nd:YAG laser. We used laser intensities in the µJ range and short exposure times, which were relatively mild as compared to those of previous studies^7^. However, we were not interested in identifying lethal illumination parameters but to study cellular changes after laser irradiation.

Tiina Karu in 1993 has reported series of studies using lasers at wavelengths in the 240–300-nm range. They reported that a 266-nm laser could increase cell proliferation and DNA synthesis. They tried to kill HeLa cells by continuous 271-nm and 266-nm laser irradiation, but the cells were noted to increase population and DNA synthesis in some situations^7^. The experiments did not succeed in terms of killing cancer cells, but there was some interesting results in influencing DNA. The use of photo dynamics to kill the cancer cells at longer wavelengths, such as from 630–762 nm, was more successful^6^. Further work on the effect of 266-nm light subsequently stalled^7^.

The goal of this study was to determine if a 266-nm laser can influence protein expression, even though the wavelength was not in the protein absorption spectrum^5^. We chose nine proteins to look for any positive result (Supplementary Table 1). CD24, CD34, CD44v6, CD45, CD73, CD90, and CD95*,* were selected owing to their availability, and CD105 and CD279 were selected because of their involvement in cell death, as we predicted the cells might be killed by the initial irradiation. The power and exposure time of 266-nm laser were relatively mild to avoid large scale killing of the cells. The cells were divided into five groups, one control and four for the four irradiation conditions, and then flow cytometry was used to check the protein markers. Only CD90 was found obviously downregulated at an intensity of 20 μJ for 10 s (Fig. 2).

We next repeated the experiment and expanded the range of intensities and found that CD90 was downregulated from 14.6–28.8% (Fig. 3, Supplementary Table 2). We noted that power (P) and exposure time (T) were the two major factors in the energy (E) transmitted to the cells. Analysis of the data indicated that the maximal effect occurred at 30 μJ for 2 s not at 40 μJ for 10 s, meaning that if E was too high, there would not be stronger effect. There still was mild response at 5 μJ for 2 s, although we do not observe an obvious change in Fig. 2. There seems to be a precise reaction according to the energy used, but we were unable to determine a formula that demonstrates the relationship based on the current data.

When a cell is illuminated, water, gases, lipids, carbohydrates, minerals, proteins, and nucleic acids in the cell are simultaneously irradiated. Although light can be absorbed by proteins, the absorption spectrum is not around 266 nm^5^. It has been reported that although 266-nm light may cleave disulphide bonds, the bonds will reform in less than 0.1 s^9^. As our experiment was carried out over 24 h, the transient effect on proteins was excluded. DNA has peak absorption around 260 nm^3^ and is more likely to be influenced by light at 266 nm, the wavelength used in this study. (Fig. 1). Karu observed increased DNA synthesis and cell proliferation after long exposures at 266-nm and 271-nm wavelengths. However, our results did not have direct evidence that the 266-nm laser influenced DNA, so we have only presented the influence in protein expression.

Although we did not focus on DNA effects, the decrease in CD90 expression is still noteworthy, as it suggests that light either did not activate CD90 or that its expression may have been increased. Thus, other genes or factors are likely responsible for downregulating CD90, and these factors must be activated during laser irradiation. We also assessed the A549 cell line, which shows high CD90 expression. Irradiation with the 266-nm laser did not affect CD90 expression in A549 cells (Supplementary Fig. 2), perhaps because the factors downregulating CD90 differ between A549 cells and SDSCs. This also suggests that CD90 downregulation is cell specific and not protein specific.

The properties of light, such as wavelength, pulse width, frequency, power strength, and exposure time, in previous studies differ from those in our study^7^. Increasing the power from 30 to 40 μJ decreased the effect on CD90 expression from 28.8 to 22.0%, suggesting that power is a crucial factor in this effect. The laser power used in some previous studies ranged from millijoules to joules^7^, that is, 10^3^–10^6^-fold greater than the intensity used in our studies. As the goals of the previous studies were to kill cancer cells, we can conclude that adequate light intensity is an important factor in the effect of light on cells.

This innovative study found that the 266-nm light altered protein expression in a stem cell line. This effect is cell specific, repeatable, and dependant on the light intensity. The phenomenon could be used to change cellular function through protein alteration. It is the first report that a specific protein in SDSCs can be changed by 266-nm light, and further studies are required to develop a potential practical use.

## Materials and methods

### Nucleotides

Adenine, thymine, cytosine, guanine, and uridine were purchased from Sigma-Aldrich (St. Louis, MO, USA). The purity of adenine, thymine, cytosine, and uridine was ≥ 99%, whereas that of guanine was 98%. We did not perform further purification before the experiments.

### Absorption spectroscopy

A Cary 50 Spectrophotometer, Instrument Version 3.00, from Varian Company (Palo Alto, CA, USA) was used. Adenine, thymine, cytosine, guanine, and uridine were diluted to 50 μM/L, and their absorption spectra were measured over a range of 240–280 nm with 40 dividing scales for adenine, thymine, cytosine, and uridine, and with 100 dividing scales in the range of 240–290 nm for guanine.

### Laser machine and laser light

A Minilite laser was purchased from Continuum (Santa Clara, CA, USA), which is an actively Q-switched Nd:YAG laser. The laser produces horizontal polarised infrared light at 1064 nm. A non-linear crystal was placed in the beam to quadruple the frequency to an equivalent wavelength of 266 nm. A prism was used to refract the 266 nm laser directly to the target, a cross marker on a microscopy plate. The laser was detected using fluorescent paper. The laser pulse width was 5 ± 2 ns and the firing frequency was 10 Hz. The laser intensity was calibrated in μJ using a Micro Joule Meter of PEM 100 Display procured from LTB Lasertechnik (Berlin, Germany).

### Culture of skin-derived stem cells (SDSCs)

Human FB343 P:4 SDSCs were provided by Maria von Med-Biotechnology Co., Ltd. (Taipei, Taiwan). SDSCs were cultured in Dulbecco’s Modified Eagle Medium (DMEM)/High Modified from HyClone (Logan, UT, USA) at 37 °C, 5% CO_2_, and 95% humidity in a CO_2_ incubator. When the cells reached a density of 10^7^ cells/mL, they were used in the experiments.

### Cellular markers

Nine cellular markers (CD24, CD34, CD44v6, CD45, CD73, CD90, CD95, CD105, and CD279) were selected for the experiments (Supplementary Table 1). These markers were used to detect possible alterations in protein expression in SDSCs.

### Flow cytometry

Flow cytometry for SDSCs was performed using a Cytoflex Flow Cytometer (Beckman Coulter, Brea, CA, USA) at Maria von Med-Biotechnology Co., Ltd. Flow cytometry for A549 cells was conducted using a FACSCanto II Flow Cytometer (BD Biosciences, San Jose, CA, USA) at the Genomic Research Center of Academia Sinica (Taipei, Taiwan).

### Flow cytometry for cellular markers in SDSCs exposed to 266-nm laser light at four intensities

SDSCs were cultured in a CO_2_ incubator in DMEM/High Modified (HyClone). The cells were grown to a density of 10^7^ cells/mL and then divided into five groups of 10^6^ cells/mL each. After laser irradiation at the varying intensities, the SDSCs were further cultured for 24 h and harvested. The cell numbers and morphologies were determined by microscopy. To detect the expression of cell surface markers, 1 μL of antibodies specific to CD24, CD34, CD44v6, CD45, CD73, CD90, CD95, CD105, and CD279 were added, respectively, to individual tubes containing 100 μL SDSC suspensions. The tips of the tubes were flicked, and the fluorescence intensities were detected by flow cytometry.

### Detection of CD90 expression in SDSCs irradiated with 266-nm laser at seven different intensities

To detect CD90 expression in SDSCs following 266-nm laser irradiation, the cells were exposed to light at seven intensities (5 μJ for 2 s, 20 μJ for 2 s, 20 μJ for 10 s, 30 μJ for 2 s, 30 μJ for 10 s, 40 μJ for 2 s, and 40 μJ for 10 s). The cells were then examined for CD90 expression using flow cytometry, as described above.

### Culture of A549 cells

The A549 human lung cancer cell line (CCL-185, Lot number: 63913710) was purchased from the American Type Culture Collection (Manassas, VA, USA). Cells were cultured in F-12 K Medium (Kaighn's Modification of Ham's F-12 Medium) containing 2 mM l-glutamine and 1500 mg/L sodium bicarbonate at 37 °C, 5% CO_2_, and 95% humidity in a CO_2_ incubator. When the cells reached a density of 10^7^ cells/mL, they were used in the experiments.

### Detection of CD90 expression in A549 cells irradiated with a 266-nm laser at five intensities

Cells were first irradiated by the 266-nm laser at intensities of 5 μJ for 2 s, 30 μJ for 2 s, 70 μJ for 5 s. Subsequently, they were again irradiated at 30 μJ for 3 s, and 100 μJ for 10 s. CD90 expression on A549 cells was examined by flow cytometry.

### Statistical analysis

Statistical analyses were carried out using GraphPad Prism software version 6 (La Jolla, CA, USA). The *P*-values were determined using Student’s *t*-tests, and results showing *P* < 0.5 were considered statistically significant. The control mean fluorescent intensity value was considered to be 100%, and the data of the experimental groups were normalised to those of the control.

## Supplementary Information

Supplementary Information.

## Data Availability

Requests for materials should be addressed to R. L. H. (rayling.hsiao@msa.hinet.net) and C.Y.W. (cyiwu@gate.sinica.edu.tw).
