# Supplementary material for: Innovative finding of 266-nm laser regulating CD90 levels in SDSCs
Source: Sci Rep. 2021 Jul 6;11:13932. doi: 10.1038/s41598-021-93508-x (PMC8260621; doi:10.1038/s41598-021-93508-x)
Supplement: Supplementary file 1 — Supplementary Information. [file 41598_2021_93508_MOESM1_ESM.pdf]

Supplementary Information

Innovative Finding of 266-nm Laser Regulating CD90 Levels in SDSCs

Ray-Ling Hsiao[1*], Yen-Chung Chen[2], Mei-Yue Huang[2,3],

Chiang-Yun Chen[4], Yu-Wei Lin[4], Chung-Yi Wu[4*]

[1]PhD Program of Graduate Institute of Cancer Biology and Drug Discovery, China Medical University, Taichung, Taiwan and Academia Sinica, ROC; [2]Maria Von Med-Biotechnology Co., Ltd., Taipei, Taiwan; [3]Mainz Dermatologic Clinic, Taipei, Taiwan; [4]Genomics Research Center, Academia Sinica, ROC

Correspondence: rayling.hsiao@msa.hinet.net (R. L. Hsiao), cyiwu@gate.sinica.edu.tw (C.Y. Wu)

1. 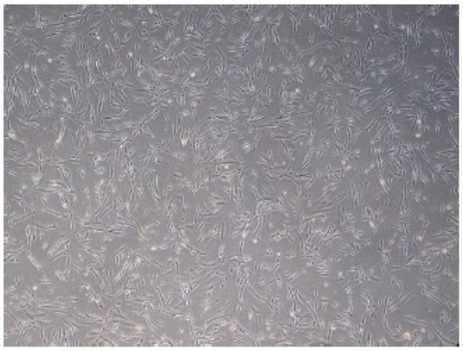

2. 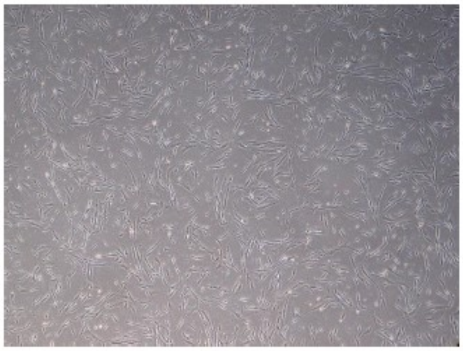

3. 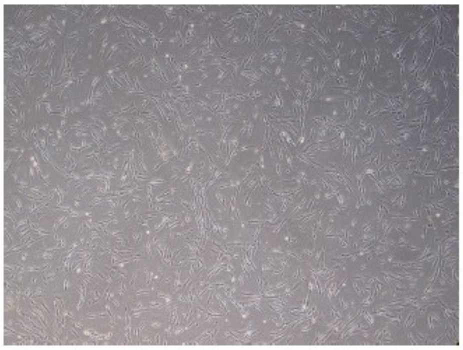

4. 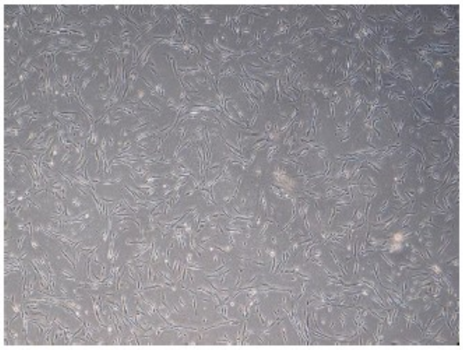

5. 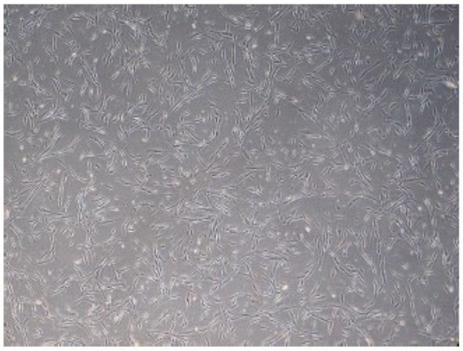

6. 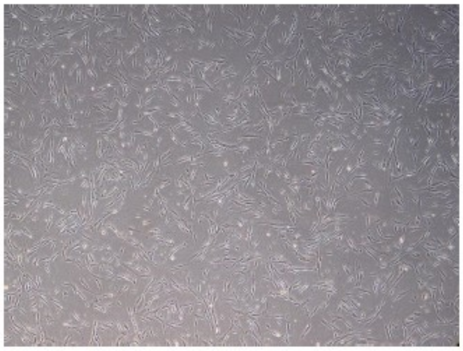

7. 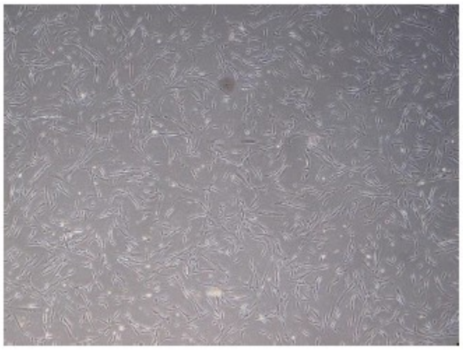

8. 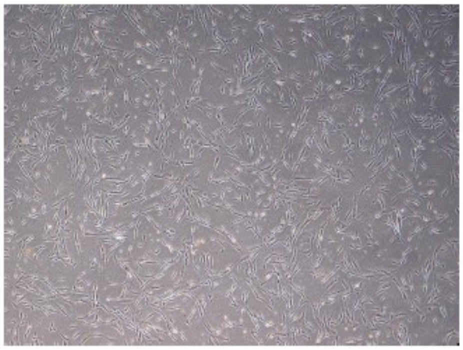

Supplementary Figure 1. Comparison of cell morphology between the control and experimental groups

Cell morphology was examined by microscopy after 24 h of culture. Panel 1 represents the control, and panels 2–8 represent cells exposed to laser light at different intensities: 5 µJ for 2 s, 20 µJ for 2 s, 20 µJ for 10 s, 30 µJ for 2 s, 30 µJ for 10 s, 40 µJ for 2 s, and 40 µJ for 10 s, for panels 2–8, respectively.

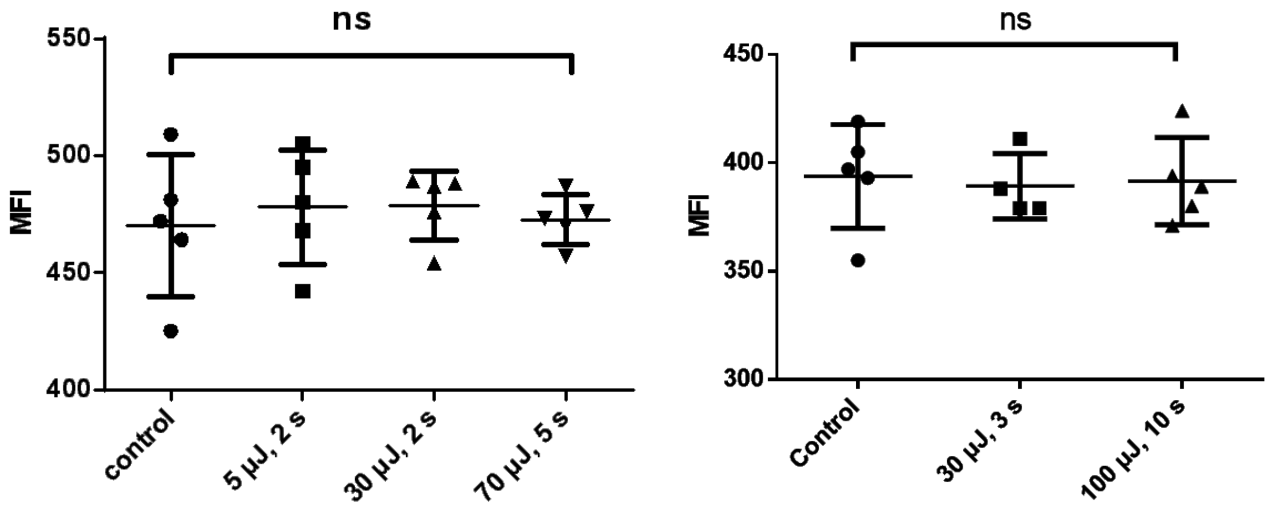

Supplementary Figure 2. Five samples of A549 lung cancer cells irradiated at five different intensities using a 266-nm laser (5 µJ for 2 s, 30 µJ for 2 s, 70 µJ for 5 s, 30 µJ for 3 s, and 100 µJ for 10 s). No significant changes in MFI were observed.

Supplementary Table 1. Cellular markers evaluated

Cell Marker	Description	Manufacturer
CD24	CD24 monoclonal antibody, PE	Thermo Fisher Scientific, Waltham, MA, USA
CD34	FITC anti-human CD34 antibody	Biolegend, San Diego, CA, USA
CD44v6	Human CD44v6 PE-conjugated antibody	Bio-techne, Minneapolis, MN USA
CD45	PE anti-human CD45 antibody	Biolegend, San Diego, CA, USA
CD73	FITC anti-human CD73 antibody	Biolegend, San Diego, CA, USA
CD90	PE anti-human CD90 (Thy1) antibody	Biolegend, San Diego, CA, USA
CD95	PE mouse anti-human CD95 antibody	BD Biosciences, San Jose, CA, USA
CD105	FITC anti-human CD105 antibody	Biolegend, San Diego, CA, USA
CD279	PE mouse anti-human CD279 antibody	BD Biosciences, San Jose, CA, USA

Supplementary Table 2. Comparison of fluorescence intensities between the control and experimental groups using flow cytometry

Tube Name	Panel	Laser intensity	Mean PE-H	Regulation rate
1-2-CD90	1	0	307,145.6	0%
2-2-CD90	2	5 μJ, 2 s	260,889.6	–15.1%
3-2-CD90	3	20 μJ, 2 s	262,928.6	–14.6%
4-2-CD90	4	20 μJ, 10 s	232,058.9	–24.4%
5-2-CD90	5	30 μJ, 2 s	218,592.5	–28.8%
6-2-CD90	6	30 μJ, 10 s	224,002.8	–27.1%
7-2-CD90	7	40 μJ, 2 s	239,722.8	–22.0%
8-2-CD90	8	40 μJ, 10 s	231,954.0	–24.5%

Panel 1 represents the control group, and Panels 2–8 represent cells subjected to different laser intensities and then cultured for 24 h. Panel 2: 5 μJ for 2 s; Panel 3: 20 μJ for 2 s; Panel 4: 20 μJ for 10 s; Panel 5: 30 μJ for 2 s; Panel 6: 30 μJ for 10 s; Panel 7: 40 μJ for 2 s; and Panel 8: 40 μJ for 10 s.

Supplementary Table 3. Comparison of cell populations between the control and experimental groups

SDSCs were irradiated with 266-nm laser light. Cells were enumerated after 24-h culture. Data are expressed as $\times 10^6$ cells/mL.

Sample	1	2	3	4	5	6	7	8
Laser intensity	0	5 μJ, 2 s	20 μJ, 2 s	20 μJ, 10 s	30 μJ, 2 s	30 μJ, 10 s	40 μJ, 2 s	40 μJ, 10 s
Cell population	1.161	1.145	1.195	1.126	1.267	1.242	1.214	1.175
